# Alveolar organoids: from model systems to applications in respiratory diseases

**DOI:** 10.1186/s12967-026-08616-x

**Published:** 2026-07-22

**Authors:** Yuanfang Xing, Luyu Yang, Zhimin Cao, Dongchang Wang, Huan Ye

**Affiliations:** 1 Beijing Tuberculosis and Thoracic Tumor Research Institute, Courtyard 1, No.9 Beiguan Street, Tongzhou District, Beijing, 101199 China; 2 Department of Respiratory and Critical Care Medicine, Beijing Chest Hospital, Capital Medical University, Courtyard 1, No.9 Beiguan Street, Tongzhou District, Beijing, 101199 China; 3 Department of Respiratory and Critical Care Medicine, Peking University Third Hospital, Peking University, Beijing, 100191 China

**Keywords:** Organoid, Alveolar organoid, Respiratory disease, Three-dimensional culture

## Abstract

Organoid technology has emerged as a research hotspot in the biomedical field in recent years, providing important methodologies for disease modeling, drug screening, and regenerative medicine. Alveolar organoids serve as crucial models for studying respiratory diseases, and their technological evolution and application expansion reflect key advances in this field. This article systematically reviews the developmental history of organoids, provides an in-depth comparison of the applicability and limitations of different culture and characterization strategies for alveolar organoids, summarizes and critically analyzes alveolar organoid models established for common respiratory diseases, and offers perspectives on future directions in this area.

**Supplementary Information:**

The online version contains supplementary material available at https://doi.org/10.1186/s12967-026-08616-x.

## Search strategies

The core references for this review were systematically identified through subject searches conducted in the PubMed/Medline, Web of Science core databases, and Embase databases, covering the period from January 1, 2009 to the present (specific search strategies are detailed in Supplementary Table 1).

**Supplementary Information:**

The online version contains supplementary material available at https://doi.org/10.1186/s12967-026-08616-x.

## Overview and developmental history of organoids

Since the early 20th century, two-dimensional (2D) cell culture technology has been a cornerstone of in vitro experiments, playing a vital role in scientific research. However, 2D models exhibit deviations in characterizing tissue cells within an in vitro environment and possess numerous limitations [1–3]. As an alternative, three-dimensional (3D) organoid culture technology demonstrates significant advantages in morphological observation, cell population monitoring, proliferation capacity assessment, response to stimuli analysis, differentiation potential studies, drug metabolism analysis, and protein synthesis experiments, with its application scope gradually expanding [4–7].

In 3D organoid culture experiments, the cellular microenvironment can be manipulated to mimic the in vivo environment, thereby providing more precise data for studying cell-cell interactions, tumor characteristics, drug discovery, metabolic analysis, stem cell research, and other disease types [6, 8, 9]. During experiments, 2D models remain irreplaceable for initial drug target screening and basic studies of cellular metabolism due to their homogeneity and high-throughput capabilities [10]. The core advantage of 3D organoids lies in their ability to reconstruct in vivo-like cell polarity and paracrine signaling gradients, thereby recapitulating specific cellular behaviors observed in vivo [11]. However, this complexity also imposes higher technical demands and greater economic costs. Therefore, researchers should make trade-offs and select the most appropriate approach based on their specific scientific questions [12]. Table 1 compares the different aspects of 2D and 3D cell cultures and elaborates on the advantages and disadvantages of both methods.

**Table 1:** Comparison between 2D cell culture and 3D organoid culture

	Cells	Organoids
Growth Environment	Grown on flat culture dishes or flask surfaces as a monolayer	Grown in 3D scaffolds, gels, or matrices, allowing spatial expansion and interaction
Cell Morphology	Typically flattened	More three-dimensional and natural morphology, capable of forming tissue-like structures
Survival Rate	High	Low
3D Growth Capability	Low	High
Cost	Low, suitable for large-scale experiments	High, suitable for specific research needs
Operational Complexity	Simple	Relatively complex
Resource Consumption	Low cost, suitable for large-scale experiments	High cost, with higher demands for operation and maintenance
Mimicry of In Vivo Environment	Low	High
Cell-Cell Interactions	Occur in a two-dimensional plane, limited interactions	Complex, closer to in vivo growth state
Proliferation Capacity	May differ from in vivo conditions	Closer to actual in vivo conditions
Disease Model Construction	Difficult to construct complex disease models	Can construct disease models closer to in vivo conditions, such as cancer and neurodegenerative disease models
Applications	Suitable for basic cell biology research, large-scale cell expansion, and high-throughput screening	Widely used in tissue engineering, regenerative medicine, disease model construction, and drug screening

**Fig. 1:**
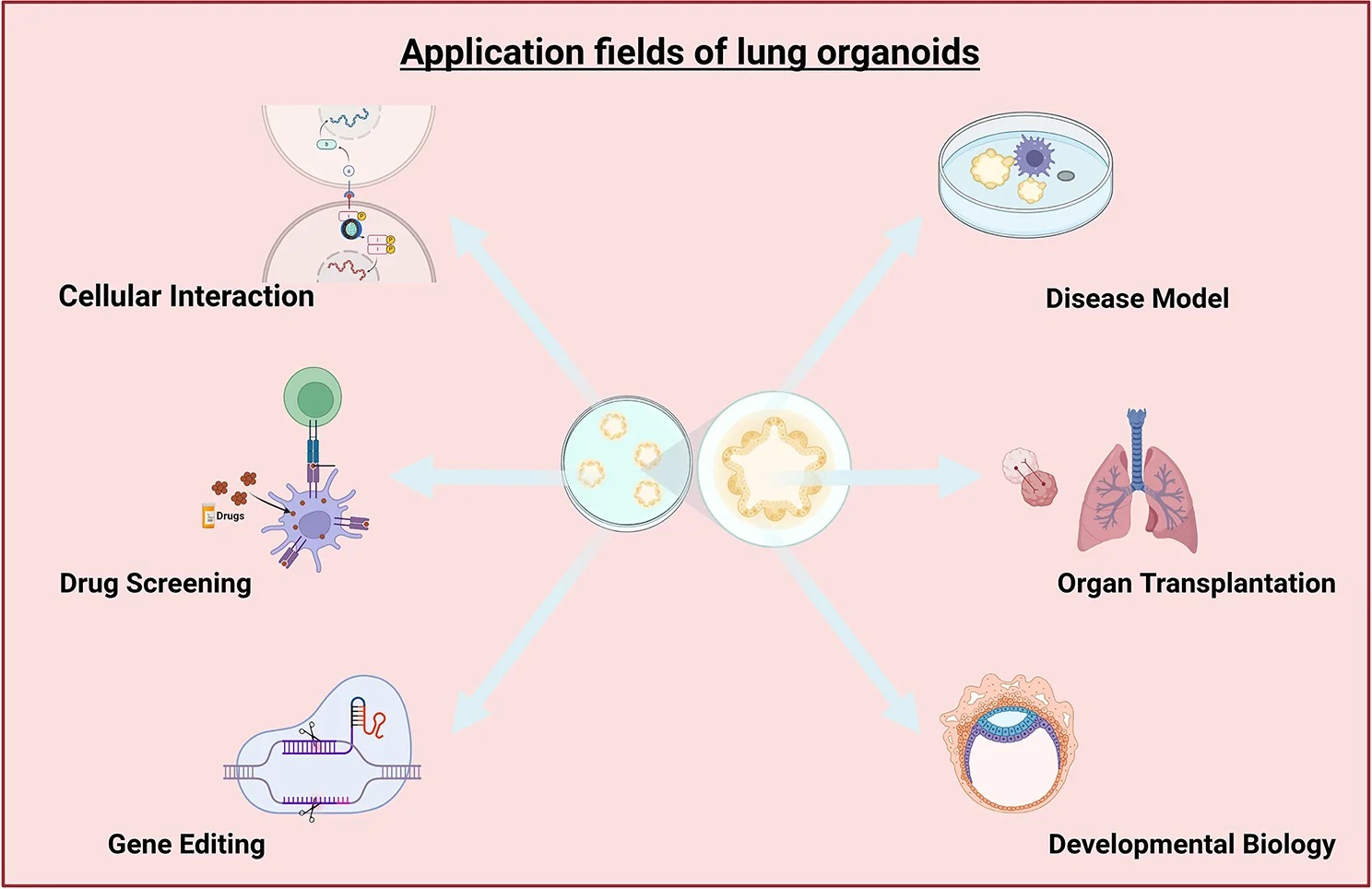
Application fields of lung organoids

Organoid technology involves three-dimensional miniature cell clusters. By inducing the division and differentiation of progenitor cells such as induced pluripotent stem cells (iPSCs), embryonic stem cells (ESCs), or adult stem cells (ASCs) in vitro, these clusters can form 3D in vitro models that contain the same cell types as the original organ and possess self-renewal and self-organization capabilities. This technology can closely mimic the structure and function of corresponding organs in vivo, holding significant value in biomedical research, disease model construction, drug screening, and personalized medicine [13–15]. Figure 1 illustrates the main application areas of organoid technology in recent years.

## Respiratory diseases and the development of alveolar organoids

The respiratory system is one of the vital physiological systems in the human body. It controls lung expansion and contraction through the contraction and relaxation of respiratory muscles such as the diaphragm and intercostal muscles, enabling the respiratory movements of oxygen intake and carbon dioxide exhalation. Additionally, the respiratory system plays important roles in thermoregulation, vocalization, and pathogen defense. With increasing air pollution and the growing smoking population, the prevalence of chronic respiratory diseases, primarily chronic obstructive pulmonary disease (COPD), continues to rise globally. Between 1990 and 2019, global deaths and confirmed cases due to chronic respiratory diseases increased by 28.5% and 39.8%, respectively [16]. Although existing treatment strategies include various approaches such as pharmacotherapy, oxygen therapy, pulmonary rehabilitation, and lifestyle modifications, their efficacy remains limited, underscoring the urgent need for basic research to explore more effective treatment options.

Alveoli are the basic units of gas exchange, primarily composed of alveolar type I (ATI) and alveolar type II (ATII) epithelial cells. By inducing stem cells to differentiate into ATI/ATII cells to form alveolar organoids, vesicular structures mimicking the sac-like structure of alveoli can be constructed, possessing functions such as an air-liquid interface, surfactant secretion, and damage repair [17–19].

In 2009, Rock and colleagues discovered that basal cells in the mouse and human airways could serve as stem cells for airway epithelial cells and successfully cultivated “tracheospheres” using in vitro culture techniques [20]. In 2013, the Barkauskas team demonstrated that alveolar epithelial stem cells could differentiate into “alveolospheres,” laying the foundation for constructing alveolar organoids [21]. In 2015, the Dye research team utilized human pluripotent stem cells (hPSCs) to construct lung organoid models featuring proximal airway structures and distal epithelial cells, exhibiting structural characteristics highly similar to human fetal lung tissue [22]. In the same year, the Tan team successfully constructed airway organoids using primary human bronchial proximal epithelial cells, lung fibroblasts, and pulmonary microvascular endothelial cells [23].

In recent years, with the continuous development of organoid technology, researchers have actively adopted alveolar organoid models to simulate human lung physiology and pathology, achieving a more accurate recapitulation of the human lung microenvironment. This provides crucial theoretical foundations and experimental data supporting clinical research and the development of treatment strategies.

## Methods for culturing and characterizing alveolar organoids

### Alveolar organoid culture methods

The choice of culture strategy for alveolar organoids requires a balance between two core dimensions: biological fidelity and experimental operability, using ATII cells as seed cells for organoid culture [17, 24]. ATII cells are typically isolated from lung tissue using techniques such as fluorescence-activated cell sorting (FACS) or magnetic-activated cell sorting (MACS). The isolated ATII cells are then cultured in support systems like Matrigel [21, 25]. Compared with traditional air-liquid interface (ALI) culture, alveolar organoid culture systems possess unique advantages in simulating the physiological and pathological microenvironment of the lung [26]. ALI culture is more suitable for short-term studies of epithelial barrier function, offering advantages such as ease of operation, low cost, and the ability to monitor transepithelial electrical resistance (TEER) in real time. In contrast, 3D organoid culture is more appropriate for long-term studies of alveolar development, injury repair, and cell-cell interactions. The dense 3D matrix environment requires neutrophils to actively remodel the extracellular matrix and squeeze through epithelial gaps, thereby more faithfully recapitulating the alveolar infiltration process during sepsis and chronic infection [27–29]. More importantly, within the enclosed 3D alveolar space, alveolar macrophages (AMs) and ATII epithelial cells establish sustained paracrine interactions, maintaining their dual role as “gentle protectors of the epithelium”—clearing pathogens through pro-inflammatory responses while promoting epithelial repair via anti-inflammatory signals [28, 30–32].

However, a key issue in current research is that most studies lack a clear rationale for their choice of method, often defaulting to the Matrigel embedding approach without fully considering the impact of different matrices on the differentiation efficiency of ATII cells toward ATI cells. Studies have shown that although Matrigel contains various growth factors and can mimic the structural, physical, and functional properties of the in vivo basement membrane [33, 34], the high abundance of BMP signaling in Matrigel may accelerate differentiation while reducing proliferative capacity. In contrast, synthetic hydrogels can more precisely replicate the mechanical properties of the alveolar basement membrane by modulating elastic modulus and have been reported for use in organoid culture [34–36]. Additionally, these alternative materials can overcome batch-to-batch variability in composition and performance associated with Matrigel, reduce heterogeneity in organoid size and structure, and show broad application prospects [37]. Other alternative materials have also been shown to overcome batch-to-batch variability in Matrigel composition and performance, reduce heterogeneity in organoid size and structure, and demonstrate promising potential for application [30, 38, 39]. Therefore, it is recommended that future studies clearly state the rationale for matrix selection in the methods section and report the inter-batch coefficient of variation.

### Methods for characterizing alveolar organoids

**Fig. 2:**
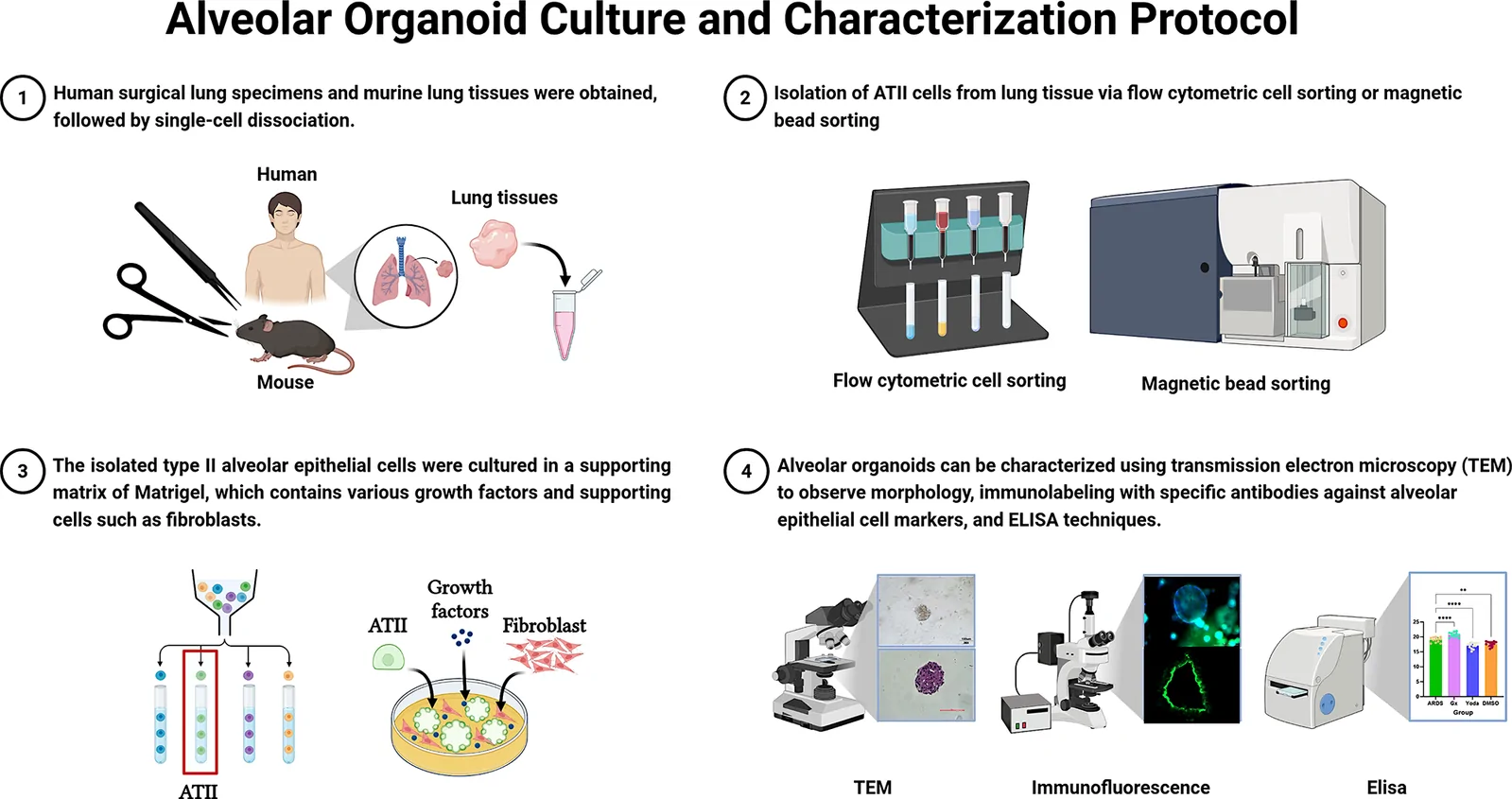
Process for lung organoid culture and identification

Methods for characterizing alveolar organoids are similar to those used for cell identification. Mature alveolar organoids typically exhibit spherical or cystic structures, which can be observed via transmission electron microscopy or scanning electron microscopy to verify their morphological similarity to native alveoli (Fig. 2).

Genetic similarity can be assessed using real-time quantitative PCR (RT-qPCR) or single-cell RNA sequencing techniques, evaluating the expression levels of genes such as surfactant protein C (SP-C), surfactant protein A (SP-A), and CC10 to determine consistency with native alveolar tissue characteristics [40–43]. The differentiation status of organoids can be evaluated using specific antibodies to label alveolar epithelial cell markers: for example, verifying the presence of ATII cells in alveolar organoids by detecting SP-C, and confirming the presence of ATI cells by detecting AQP5 [41, 44, 45]. Mimicking physiological functions is a critical prerequisite for experimental relevance. Assessing the physiological functions of alveolar organoids can include simulating viral infections (e.g., SARS-CoV-2 infection) to evaluate immune responses and cellular functions [46–48]. Following the successful establishment of relevant pathological models, validation is required using parameters such as morphological similarity [49], inflammation levels [49], and changes in cell permeability [50]. However, current literature shows some inconsistency in the criteria used to determine organoid maturity. Some studies rely solely on SP-C expression to confirm ATII cell identity, whereas more rigorous standards require simultaneous verification of lamellar body presence and surfactant secretion function [51, 52]. This lack of standardized criteria makes it difficult to directly compare results across different studies. We recommend that the characterization of alveolar organoids follow a three-tiered validation approach at the morphological, molecular, and functional levels: the first tier involves clear observation of cystic structures under light microscopy and lamellar bodies under electron microscopy; the second tier includes co-expression of ATI and ATII markers and single-cell transcriptomic lineage tracing [53]; and the third tier comprises functional responses to injury stimuli and quantitative assessment of surfactant secretion [54, 55].

## Application of alveolar organoids in research on common respiratory diseases

### Infectious respiratory diseases

#### Pneumonia

Pneumonia is a common lower respiratory tract infection that imposes a significant disease burden and economic costs globally, representing a major public health issue requiring urgent resolution. The severity of symptoms during the acute phase of this disease varies; infection with certain pathogens can lead to sputum of specific colors, and the disease is highly contagious. Bacterial pneumonia is often accompanied by high fever and chills, while viral pneumonia tends to occur seasonally [56]. According to the World Health Organization (WHO), lower respiratory tract infections caused approximately 2.6 million deaths in 2019, ranking as the fourth leading cause of death globally [57, 58].

##### Bacterial pneumonia

The most common pathogens causing bacterial pneumonia include Streptococcus pneumoniae, Haemophilus influenzae, and Legionella species, typically presenting with severe symptoms. In recent years, due to increased vaccination rates and the judicious use of antibiotics, the incidence and mortality of bacterial pneumonia have decreased in some regions. However, the emergence of drug-resistant strains remains a major challenge, particularly in developing countries [59].

Lipopolysaccharide (LPS), a major component of the outer membrane of Gram-negative bacteria composed of lipids and polysaccharides, is an important endotoxin that can induce pathological reactions such as fever, disseminated intravascular coagulation, and multiple organ failure. LPS binds to its primary receptor, Toll-like receptor 4 (TLR4), to trigger downstream signaling pathways, activating inflammatory pathways such as NF-κB and thereby inducing a severe inflammatory response [60]. Therefore, LPS is commonly used to model alveolar damage and acute respiratory distress syndrome (ARDS) [45]. Notably, although simple two-dimensional air-liquid interface (ALI) culture can support epithelial cell differentiation, it has limitations in simulating the three-dimensional developmental characteristics of the lung and complex cell-cell interactions [26]. In contrast, three-dimensional lung organoid models provide a multicellular topological structure closer to the in vivo environment, recapitulating alveolar development and cell-cell interactions, which enables TLR4-mediated signaling to exhibit more complex regulatory patterns in space, thereby potentially reflecting LPS-induced inflammatory responses more authentically. Zhu and colleagues constructed alveolar organoids using mouse lung tissue, induced an acute lung injury (ALI) model with LPS, and co-cultured them with mesenchymal stem cells (MSCs). The study found that co-culture inhibited the LPS-induced inflammatory response and suppressed alveolar macrophage inflammation and phenotypic switching via the NF-κB pathway, offering a new perspective for ALI treatment [61]. This three-dimensional organoid environment better mimics the enclosed pulmonary microenvironment than traditional ALI models, thereby enabling more accurate assessment of the dual function of AMs as “gentle protectors of the epithelium.” Swart et al. utilized organoids derived from human bronchial epithelial cells to successfully simulate the process by which Pseudomonas aeruginosa invades through goblet cells and disrupts the epithelial barrier, revealing multiple virulence factors of this bacterium [62]. Such three-dimensional models provide a more authentic platform for investigating the mechanisms of bacterial barrier breach and for assessing neutrophil chemotaxis and responses within the confined organoid space [63]. Additionally, some researchers have used LPS-induced ARDS alveolar organoid models for screening and evaluating potential therapeutic drugs [64, 65].

##### Viral pneumonia

Common pathogens of viral pneumonia include influenza virus, respiratory syncytial virus, and coronaviruses. During viral infection, the host induces the production of IFN-λ (type III interferon) and other immune defense factors to restrict viral replication and protect respiratory epithelial cells [66–68]. Symptoms often present as influenza-like illness, and the epidemiology shows distinct seasonality. During the COVID-19 pandemic, global prevention and control measures (such as lockdowns and vaccination) significantly reduced viral transmission rates and mortality. However, the emergence of novel viral variants continues to pose a threat to public health [69, 70].

Virus-related models primarily focus on SARS-CoV-2 and influenza viruses. IFN-λ serves as the first line of defense against viral infection and is preferentially produced by epithelial cells at the early stage following viral challenge. This early IFN-λ response is crucial for shaping the immune reaction and preventing the progression of severe disease [71]. However, traditional 2D culture models lack 3D tissue architecture, immune cell components, and extracellular matrix, making them insufficient to fully evaluate the protective role of IFN-λ in complex lung injury environments. In contrast, 3D lung organoid models can recapitulate the physiological exposure conditions of epithelial cells, enabling assessment of mucociliary clearance, epithelial barrier disruption, and local immune responses, thereby providing a more physiologically relevant platform for investigating the antiviral protective mechanisms of IFN-λ [71, 72]. Lung organoids can mimic alveolar structures and cell types, allowing researchers to more realistically study the viral infection process and pulmonary cell tropism. One study explored the application of human organoid models in SARS-CoV-2 infection research, particularly emphasizing their important role in simulating viral pathogenesis, lung infection, and drug development [73]. Another study employed alveolar organoid models to investigate the co-infection mechanism of SARS-CoV-2 and influenza A virus (FLUAV). The results showed that co-infection significantly enhanced the replication capacity of both viruses, leading to excessive activation of pro-inflammatory and immune-related signaling pathways and exacerbating cellular damage. This research provides crucial insights into the mechanisms underlying enhanced pathogenicity during viral co-infection and may aid in developing treatment strategies for such co-infections [74].

Although alveolar organoid models related to bacterial and viral pneumonia are widely used, these models remain relatively simplistic, lacking other tissue components such as the vascular system and neural components, which may limit the study of certain complex mechanisms in lung injury and repair. Furthermore, Matrigel may hinder the free movement of certain viruses and proteins [74].

Additionally, possibly due to the high environmental requirements of fungi, there is a lack of relevant research on fungal pneumonia models. Future research could focus on constructing fungal pneumonia models to further advance precise diagnosis, treatment, and personalized treatment options, thereby providing patients with more effective therapeutic choices [75].

#### Tuberculosis

Tuberculosis (TB) is a chronic infectious disease caused by Mycobacterium tuberculosis, characterized by symptoms such as persistent cough, sputum production, hemoptysis, low-grade fever, and night sweats. The disease severely damages lung structure and function and is a leading cause of death and disease burden worldwide [76]. Data from the past five years indicate that despite a slight decline in global TB mortality after peaking in 2021, TB remains a major global public health issue [77, 78]. Especially when tuberculosis coexists with other chronic diseases such as HIV, the threat is significantly exacerbated, as the two conditions mutually accelerate disease progression and complicate diagnosis and treatment. According to the World Health Organization (WHO) 2023 report, HIV-positive individuals accounted for 6.3% of new tuberculosis cases in 2022, and approximately 167,000 HIV-positive individuals died from tuberculosis [79, 80]. Coinfection with HIV and M. tuberculosis leads to immunodeficiency, promoting tuberculosis transmission and relapse, while M. tuberculosis also drives HIV replication and immune activation. Consequently, treatment of coinfected patients is considerably challenging, with a success rate of only 79%, lower than that of non-HIV patients [81].

Against this background, alveolar organoid models based on 3D technology demonstrate unique translational value. Alveolar organoid models based on three-dimensional technology can simulate the lung microenvironment, thereby more accurately reflecting the pathological process of M. tuberculosis infection, and have shown great potential in TB research in recent years [76, 82, 83].

Kim and colleagues established an in vitro TB infection model using human lung organoids to study host-directed therapy and the efficacy of anti-TB drugs. Their study confirmed that M. tuberculosis H37Rv could infect both lung epithelial cells and macrophages, and knocking down host genes (such as MFN2 and HERPUD1) effectively inhibited its growth, providing strong support for developing novel anti-TB therapies. Furthermore, this model allowed for long-term culture and multiple passages while maintaining M. tuberculosis viability [84].

Lung organoid models successfully recapitulated the inflammatory response and tissue damage following M. tuberculosis infection, providing a valuable tool for studying host-pathogen interactions and for drug screening and efficacy evaluation. Testing the activity of different anti-TB drugs using organoid models enables more efficient screening of effective drug combinations, potentially shortening clinical trial timelines and reducing costs [76]. Integrating tuberculosis alveolar organoid models with clinical data to provide a conceptual framework for exploring personalized treatment strategies represents an important direction for future research [76]. However, it is important to clearly recognize the current technological gaps. There is a fundamental contradiction between the turnaround time of organoid-based drug susceptibility testing and the clinical decision-making window for tuberculosis. Moreover, the drug concentration-effect relationships measured in organoids cannot be directly translated into in vivo pharmacokinetic parameters [85]. Therefore, for complex clinical scenarios such as tuberculosis-HIV co-infection, it is essential to acknowledge that bridging the current technological gaps—namely standardization of models, predictive validation, and regulatory approval—will be required before routine clinical application can be achieved [86].

### Non-communicable respiratory diseases

#### Chronic Obstructive Pulmonary Disease (COPD)

Chronic obstructive pulmonary disease (COPD) is a common chronic lung disease characterized by persistent respiratory symptoms, airflow limitation, and airway remodeling. It is a leading cause of death worldwide. Without effective intervention measures, the number of COPD cases globally is projected to increase to 592 million by 2050 [87]. As the disease progresses, progressive impairment of the repair function of alveolar epithelial cells in COPD patients promotes the development of emphysema and may lead to systemic inflammation. Therefore, identifying suitable methods for lung repair and regeneration is of great importance [88, 89]. In recent years, utilizing alveolar organoids to construct COPD models and investigate alveolar epithelial cell repair mechanisms has gained widespread recognition [88, 90–92].

Some studies construct in vitro models using epithelial cells from patients with COPD. A study using ex vivo alveolar organoid models found a significantly enriched “ATII subtype” in the alveolar sacs of COPD patients, with transcriptomic characteristics differing from normal ATII cells. This study revealed the potential impact of this “ATII subtype” on alveolar regeneration in COPD pathogenesis and identified potential targets for developing novel COPD treatment strategies [90]. Other studies construct disease models by applying common COPD inducers, such as cigarette smoke extract (CSE). Chan and colleagues established airway organoids derived from COPD patients to study host-pathogen interactions, revealing enhanced viral fitness of SARS-CoV-2 in bronchi and providing a robust platform for modeling COPD pathophysiology [93].

Obtaining cell samples from patients with COPD presents significant difficulties, and insufficient sample sizes may affect the generalizability of research findings [90]. In contrast, results obtained by treating alveolar organoids with extracts from various harmful particles are highly consistent with most in vivo and in vitro disease models and can more directly demonstrate the pathological processes of alveolar epithelial cells. Therefore, constructing COPD organoid models by co-culturing alveolar cells derived from healthy individuals with harmful particle extracts is expected to find widespread application in studying COPD pathogenesis and lung regeneration mechanisms [94].

#### Idiopathic pulmonary fibrosis

Pulmonary fibrosis is a chronic, progressive interstitial lung disease, among which idiopathic pulmonary fibrosis (IPF) has the highest mortality rate. Globally, the incidence and prevalence of IPF show significant geographical variation, with prevalence increasing with age; most patients are diagnosed over the age of 50 [95–97].

In recent years, organoid technology has been increasingly applied in IPF research. Adding transforming growth factor-β1 (TGF-β1) during alveolar organoid culture can induce a fibrotic phenotype in lung organoids resembling IPF, providing a new platform for lung disease modeling and drug screening, thereby validating its clinical translational value [98–100]. The research team led by Cohen enriched ATII cells from IPF patients to culture IPF organoids and found that epigallocatechin gallate (EGCG) alleviated IPF-related transcriptional changes and promoted ATII to basal cell metaplasia. This discovery offers a new potential therapeutic target for IPF [101].

#### Lung cancer

Lung cancer is the leading cause of cancer death worldwide, with approximately 1.8 million deaths in 2020. Non-small cell lung cancer (NSCLC) is the most prevalent type, accounting for about 85% of all lung cancer cases [102, 103]. In 2020, there were approximately 2.2 million new cases of lung cancer globally, with NSCLC representing 84%. NSCLC predominantly occurs in smoking patients, is highly aggressive, and is often associated with lymph node and distant metastasis [104, 105]. Existing treatment options include surgery, radiotherapy, chemotherapy, targeted therapy, and immunotherapy, with the specific choice requiring comprehensive assessment of the patient’s condition. However, treatment options are limited, necessitating a deeper understanding of lung cancer signaling pathways, angiogenesis, and other mechanisms at the molecular level. To improve the predictive capability of clinical responses to drug treatment, lung cancer organoid technology has been developed for high-throughput drug screening [106–109].

In an experiment investigating resistance mechanisms to KRAS inhibitors, researchers utilized alveolar organoids derived from genetically modified mice to explore the role of adenocarcinoma to squamous cell carcinoma transformation (AST). The results showed that after long-term treatment with KRAS inhibitors, these alveolar organoids exhibited pathological features of squamous cell carcinoma and developed drug resistance, revealing the mechanism of AST in KRAS inhibitor resistance and suggesting potential biomarkers for predicting resistance [110]. Although integrating versican fragment analysis, TAM polarization tracking, and matrix stiffness quantification remains technically demanding and largely confined to specialized proof-of-concept studies, emerging evidence suggests that tumor microenvironment components and immune cells critically regulate resistance. Versican deposition, for example, drives TAM M2 polarization via TLR2, fostering tumor cell plasticity. Consequently, organoid-based resistance assessment must simultaneously monitor versican fragments and TAM polarization to differentiate cell-autonomous from microenvironment-mediated resistance. Immune-vascularized models generated by 3D bioprinting of tumor organoids with immune cells and stroma have been employed to evaluate anti-angiogenic and immunotherapeutic efficacy, recapitulating tumor-stroma-vessel-immune crosstalk and spatial TAM polarization at the invasive front [111–114]. Nevertheless, most current models fail to precisely control TAMs and stromal mechanics, including versican-induced matrix hydration and interstitial pressure changes that dysregulate TAMs through PIEZO1 [115–117]. Rigorous evaluation therefore requires live-cell confocal tracking of TAM migration and phagocytosis alongside atomic force microscopy quantification of per-organoid matrix stiffness and collagen architecture [118].

Meanwhile, it has been reported that the establishment rate for pure tumor organoids is only 17%, limiting their feasibility for clinical applications. Most organoids derived from intrapulmonary tumors are overgrown by normal airway organoids, making it difficult to obtain pure tumor organoids. Furthermore, even when tumor organoids are successfully established, existing culture systems can hardly retain TAMs infiltrated in the primary tumor and ECM with pathological characteristics. The absence of TAMs may result in misjudgment of responses to immunotherapy drugs, whereas the deficiency of ECM components such as versican may mask their promoting effects on chemoresistance and angiogenesis [119–121]. This situation can also lead to misinterpretation of drug screening results, affecting the accurate assessment of tumor drug responses [122]. Three-dimensional bioprinting technology provides support for tumor organoids, but widespread scalability has not yet been achieved due to high costs, loss of modular platform characteristics, and potential oversimplification issues that may reduce clinical predictive power. If modular vascularized kits integrating controllable ECM components and immune cells can be developed in the future to enable stable construction of such models, and if prospective multicenter trials are simultaneously conducted to incorporate microenvironmental parameters—such as TAM polarization ratios and versican abundance from organoid drug screening—into predictive models, directly compare the predictive outcomes of 3D models with actual patient treatment responses, and establish levels of clinical evidence, this will help overcome the core barrier to translating “laboratory innovations” into “clinical tools.” [114, 123, 124].

### Comparison of organoid modeling strategies for different respiratory diseases

The selection of organoid platforms, construction logic, and validation standards vary significantly across the disease models discussed above, reflecting the distinct pathological nature of each disease (Tables 2 and 3).

**Table 2:** Lung organoid platform system

Technology Platform	Application advantages	Research area	Technical bottleneck	Representative study	Reference
Stem Cell Derivative System	Unlimited sources; gene-editable	Research on Developmental Mechanisms	Maturity defect (fetal lung phenotype); Matrix gel artifacts impede viral spread	SARS-CoV-2/influenza virus co-infection model; Mycobacterium tuberculosis infection model	[73, 74, 84, 135]
Primary cell organoid system	Preservation of adult tissue cell heterogeneity	Simulating natural tissue structures	Short culture cycle (typically 2–4 weeks); limited passage capability; significant inter-batch variability	Infection mechanism of Pseudomonas aeruginosa; SARS-CoV-2 invasion kinetics; Pathological phenotypes of COPD/emphysema; Metaplasia mechanism of goblet cells	[62, 90, 93, 129, 136– [138]
Patient source system	Preservation of disease heterogeneity	Personalized drug testing	Low establishment rate (only 17% in pure tumor samples) with contamination by normal epithelium; long-term culture-induced genetic drift	Establishment of NSCLC model; Study on drug-resistant transdifferentiation of KRAS inhibitors	[110, 122, 139]
Immunococulture System	Induction of innate immune response	Investigation of Inflammation-Epithelial Interaction	Macrophages cannot cross the basement membrane. Simplified immune microenvironment (only 1–2 cell types)	MSCs alleviate LPS-induced damage by modulating macrophage phenotype; The TWEAK-Fn14 axis promotes ATII proliferation	[61, 140]
Matrix Co-culture System	Simulation of epithelial-mesenchymal signaling dialogue	Investigation of fibrosis mechanisms	Abnormal activation of fibroblasts; Alteration of natural signaling pathways in the matrix	TGFβ1/sFRP2 drives ATII metaplasia into basal cells; STAT3 pathway induces cystic organoid formation of MUC5B	[65, 141, 142]
Organoid Chip System	Real-time dynamic imaging; Mechanical force stimulation	Vascularization potential	The inward-facing top surface makes pathogen exposure difficult; The technical complexity is high.	Research on Pathological Mechanisms of Tuberculosis and Drug Screening Platform	[76]
Multi-omics Integration Platform	Unbiased gene expression analysis	In-depth Exploration of Molecular Mechanisms	Limited dynamic range under infection conditions; High sample heterogeneity; High cost	Technical consistency validation of RNA-Seq versus NanoString technology; Analysis of viral response transcriptome profiles	[143]

**Table 3:** Disease-specific research platform compatibility

Disease Field	Recommended platforms	Limited platform	Technical considerations
Acute infection (bacterial, viral)	Primary epithelial organoids (rapid 2–3 weeks)	Stem cell-derived system (insufficient maturity)	Differential infection distinctions must be identified: respiratory epithelium → primary infection; alveolar infection → hPSC-ATII
Chronic inflammation (IPF, COPD)	Matrix co-culture (fibroblast interaction)	Simple epithelial organoids (lacking fibrosis)	Matrix cells must be introduced to simulate the pathological microenvironment
immunological disease, immunopathy	Immunococulture (Macrophages/Neutrophils)	Single epithelial system (no immune response)	Fidelity of the Model Determined by Macrophage Subpopulation Selection
Cancer	Patient-derived tumor organoids (PDOs)	Normal organoids (asexual tumor cells)	The issue of normal epithelial hyperplasia must be addressed (EGFR inhibition)
Drug screening	Organ-on-a-chip (high-throughput); Stem cell-derived system (scalable)	Primary system (batch instability)	Trade-off between flux and physiological relevance

#### Differences in modeling logic: Infectious diseases (bacterial pneumonia, tuberculosis) vs. chronic non-infectious diseases (COPD, IPF)

The core challenge in modeling infectious diseases is the spatiotemporal dynamics of pathogen-host interaction. Taking tuberculosis as an example, the infection cycle of M. tuberculosis spans weeks to months, requiring long-term culture stability of organoids, which explains the increasing application of hPSC-derived systems in this field [84]. In contrast, bacterial pneumonia has an acute course and relies more on primary cell-derived organoids to preserve the functional maturity of the adult lung epithelium [125]. However, a methodological blind spot exists in current research: the standardization of pathogen exposure routes in infection models. While SARS-CoV-2 organoid studies predominantly use apical inoculation to simulate respiratory exposure, M. tuberculosis studies often employ intraluminal microinjection or basolateral inoculation. This difference is not based on pathogen biology but is driven by technical convenience, potentially introducing artificial bias into infection dynamics [126–128]. Therefore, when selecting an organoid platform for modeling infectious respiratory diseases, priority should be given to the route and duration of infection, rather than solely pursuing epithelial fidelity.

#### COPD vs. IPF: Different modeling needs under similar phenotypes

Both COPD and IPF manifest as lung structural destruction, but their organoid modeling strategies should be distinctly different. The core pathology of COPD is epithelial repair dysfunction; therefore, patient-derived ATII cell organoids best reflect the nature of the disease [90]. In contrast, the core pathology of IPF is dysregulated epithelial-mesenchymal crosstalk, making pure epithelial organoids insufficient for modeling the disease and necessitating the inclusion of stromal fibroblasts [101, 129]. This comparison reveals a key principle: when the disease driver originates primarily from intrinsic epithelial abnormalities, patient-derived epithelial organoids are the preferred choice; when the disease involves multicellular interactions, co-culture systems become indispensable [130]. This principle emphasizes that the complexity of organoids should mirror the cellular origin of the disease drivers, rather than being uniformly maximized.

#### Lung cancer: A paradigm shift from disease modeling to drug prediction

**Fig. 3:**
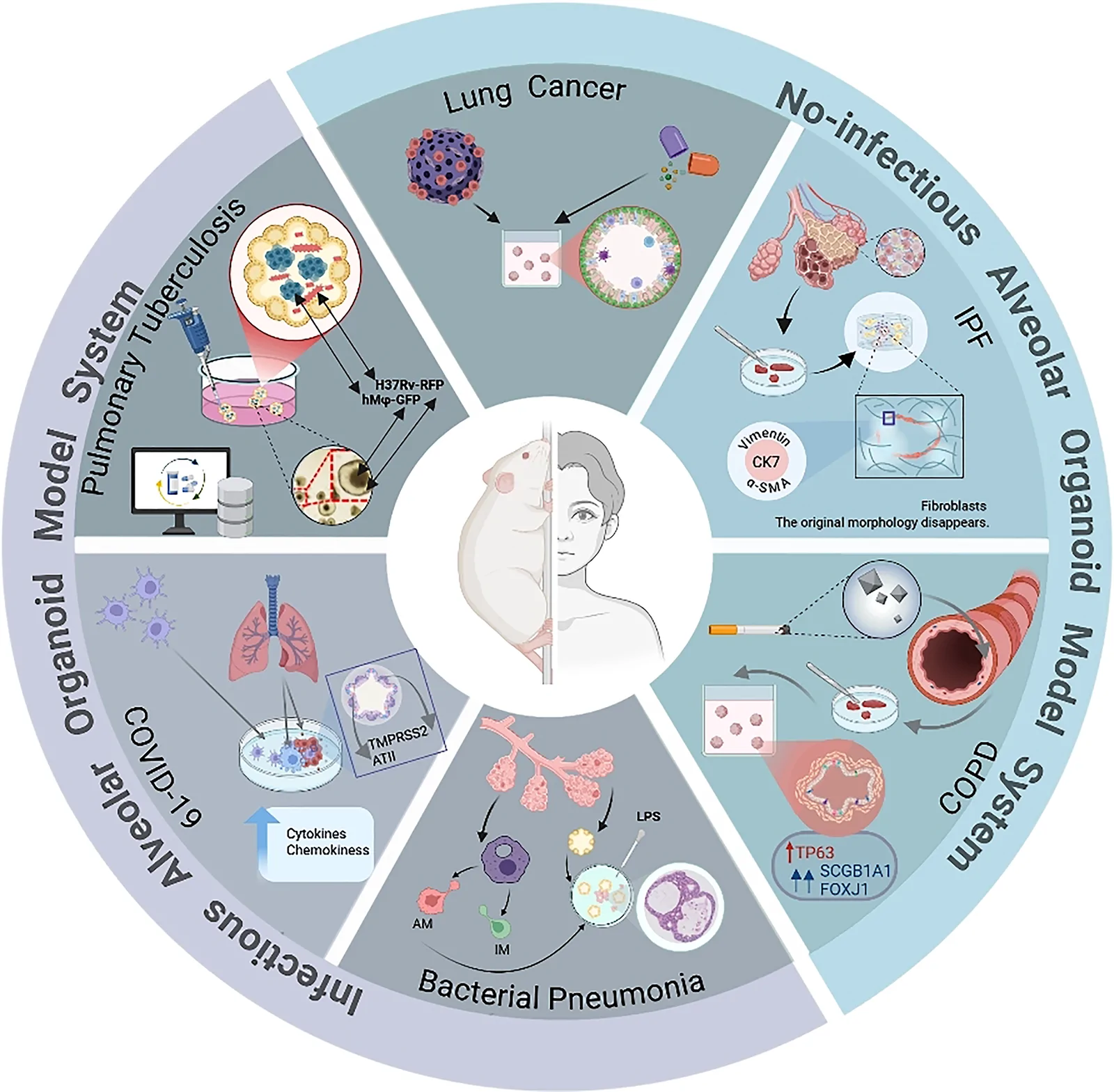
Major disease models of lung organoids

Unlike other diseases, the core objective of lung cancer organoid research has shifted from mechanistic exploration to clinical translation. This demands that models meet two additional criteria: first, retention of tumor microenvironment features, as resistance mechanisms to KRAS inhibitors involve astrocytic transformation and immune modulation via TAM polarization [110, 114, 131]; second, establishment of a predictive correlation with clinical responses [132]. However, the current establishment rate of pure tumor organoids, reported at 17%, severely constrains this translational direction [122]. In contrast, although tuberculosis organoids face similar challenges in establishment rates, their research objectives are less demanding in terms of sample size. This disparity suggests that the success rate of disease organoid models should not be evaluated by a single standard; rather, differentiated acceptable thresholds should be set according to research objectives [133]. Consequently, lung cancer organoid research needs to transition from merely replicating histological features toward building novel, predictable microenvironments—a new research paradigm (Fig. 3).

## Challenges and future perspectives

Organoid technology can bridge the gap between traditional two-dimensional cell culture and animal models, marking a significant turning point in biomedical research and opening unprecedented avenues for understanding disease mechanisms, thereby enhancing therapeutic potential for clinical applications. Lung organoid models, by simulating the physiological environment of the respiratory system, particularly when combined with bioprinting technology to construct free-form vascular networks, provide crucial support for developing personalized models for patients with various respiratory diseases. This article reviews common culture methods for lung organoids and frequently used lung organoid models for respiratory diseases.

Although significant progress has been made in using lung organoids to model lung physiology and pathology, numerous challenges remain on the path toward standardized research tools and clinical translation platforms. First, it is difficult to recapitulate the complex cellular network within the lung. Current models primarily simulate the alveolar epithelium but lack key cell types such as alveolar macrophages and fibroblasts, as well as their interactions, which limits their ability to accurately model physiological and pathological processes such as injury and repair [45]. Second, reproducibility is affected by multiple sources of variability, including batch-to-batch differences in matrices, heterogeneity in cell sources, and operator-dependent factors, yet relevant technical parameters are generally underreported in the literature [37]. Third, differences in organoids generated across different platforms may confound technical noise with true biological individual heterogeneity. Finally, a critical translational gap is the lack of clinical predictive validation for organoid-based drug screening results. Because organoids cannot simulate in vivo pharmacokinetics or systemic toxicity, their role should be positioned as a “prioritization tool for candidate drugs” rather than as a definitive model for predicting clinical efficacy [134].

Future development trends in alveolar organoid technology include integrating the immune system and circulatory system into the organoid construction process to develop multi-system models closer to real organs. Meanwhile, establishing standardized immune cell-alveolar organoid co-culture platforms for dynamic assessment of the pulmonary immune microenvironment will bridge the gap between current organoid models and the real lung immune microenvironment, thereby enabling the study of complex disease mechanisms. By combining organoid technology with cutting-edge techniques such as gene editing, artificial intelligence, and high-throughput sequencing, the application potential of lung organoids in disease modeling, drug screening, and personalized medicine will be further enhanced, providing profound new insights into complex disease research.

## Conclusion

In summary, alveolar organoids have evolved from simple “alveolar spheres” into sophisticated model systems capable of simulating specific disease phenotypes and evaluating drug responses. Their core value lies in fully meeting the modeling requirements for various respiratory disorders: infectious diseases require prioritization of spatiotemporal dynamics of pathogen exposure, while chronic diseases necessitate preservation of intrinsic epithelial repair defects. Currently, the primary bottleneck in this field is not merely technical complexity but rather the absence of standardized frameworks—including criteria for matrix selection, protocols for model validation, and evidence-based establishment of predictive validity between organoid drug sensitivity results and clinical outcomes. With future technological advancements and standardization, integrating organoid phenotypes with real-world patient data through multicenter prospective studies will enable alveolar organoids to transition from a “supplementary tool for mechanistic research” to a genuine “decision-making platform for precision medicine.”

## Supplementary Information

Below is the link to the electronic supplementary material.

Supplementary Material 1

## Data Availability

The data that support the findings of this study are available from the corresponding author upon reasonable request.
