# Supplementary material for: Alveolar organoids: from model systems to applications in respiratory diseases
Source: J Transl Med. 2026 Jul 22;24:1320. doi: 10.1186/s12967-026-08616-x (PMC13681799; doi:10.1186/s12967-026-08616-x)
Supplement: Supplementary file 1 [file 12967_2026_8616_MOESM1_ESM.docx]

| Subject | Search Strategies |
| --- | --- |
| Organoids | #1 ("organoid*"[Title/Abstract] OR "alveolosphere*"[Title/Abstract] OR "assembloid*"[Title/Abstract] OR "organotypic"[Title/Abstract] OR "spheroid*"[Title/Abstract] OR "pulmonary organoid*"[Title/Abstract] OR "organoid culture"[Title/Abstract] OR "organoid model*"[Title/Abstract]) |
| Respiratory system | #2 ("lung"[Title/Abstract] OR "pulmonary"[Title/Abstract] OR "alveolar"[Title/Abstract] OR "alveoli"[Title/Abstract] OR "airway*"[Title/Abstract] OR "bronchioalveolar"[Title/Abstract] OR "bronchi*"[Title/Abstract] OR "bronchial"[Title/Abstract] OR "trachea*"[Title/Abstract] OR "respiratory epithelium"[Title/Abstract] OR "respiratory tract"[Title/Abstract] OR "respiratory system"[Title/Abstract] OR "distal airway"[Title/Abstract]) |
| Cell sources | #3 ("pluripotent stem cell*"[Title/Abstract] OR "induced pluripotent stem cell*"[Title/Abstract] OR "iPSC*"[Title/Abstract] OR "stem cell*"[Title/Abstract] OR "cell differentiation"[Title/Abstract] OR "alveolar type 2 cell*"[Title/Abstract] OR "AT2 cell*"[Title/Abstract] OR "type II alveolar cell*"[Title/Abstract] OR "lung stem cell*"[Title/Abstract] OR "basal cell*"[Title/Abstract] OR "club cell*"[Title/Abstract] OR "distal airway stem cell*"[Title/Abstract] OR "alveolar epithelial cell*"[Title/Abstract] OR "mesenchymal stem cell*"[Title/Abstract] OR "MSC"[Title/Abstract] OR "embryonic stem cell*"[Title/Abstract] OR "ESC"[Title/Abstract] OR "goblet cell*"[Title/Abstract] OR "ciliated"[Title/Abstract] OR "cilia*"[Title/Abstract]) |
| Culture techniques | #4 ("cell culture technique*"[Title/Abstract] OR "tissue culture"[Title/Abstract] OR "3D culture"[Title/Abstract] OR "three-dimensional culture"[Title/Abstract] OR "tissue engineering"[Title/Abstract] OR "cellular spheroid*"[Title/Abstract] OR "coculture technique*"[Title/Abstract] OR "co-culture technique*"[Title/Abstract] OR "extracellular matrix"[Title/Abstract] OR "ECM"[Title/Abstract] OR "microfluidic*"[Title/Abstract] OR "microfluidics"[Title/Abstract] OR "organoid culture"[Title/Abstract] OR "air-liquid interface"[Title/Abstract] OR "organ-on-chip"[Title/Abstract] OR "lung-on-chip"[Title/Abstract] OR "bioprinting"[Title/Abstract] OR "hydrogel*"[Title/Abstract] OR "Matrigel"[Title/Abstract] OR "co-culture"[Title/Abstract] OR "coculture"[Title/Abstract] OR "bioengineer*"[Title/Abstract] OR "transwell"[Title/Abstract] OR "self-assembly"[Title/Abstract] OR "synthetic hydrogel*"[Title/Abstract]) |
| Applications | #5 ("animal disease model*"[Title/Abstract] OR "biological model*"[Title/Abstract] OR "disease model*"[Title/Abstract] OR "preclinical drug evaluation"[Title/Abstract] OR "preclinical drug test*"[Title/Abstract] OR "drug screen*"[Title/Abstract] OR "drug test*"[Title/Abstract] OR "drug response"[Title/Abstract] OR "high-throughput screening"[Title/Abstract] OR "HTS"[Title/Abstract] OR "personalized medicine"[Title/Abstract] OR "precision medicine"[Title/Abstract] OR "regenerative medicine"[Title/Abstract] OR "tissue engineering"[Title/Abstract]) |
| Infectious respiratory diseases | #6 ("COVID-19"[Title/Abstract] OR "COVID19"[Title/Abstract] OR "SARS-CoV-2"[Title/Abstract] OR "influenza"[Title/Abstract] OR "human influenza"[Title/Abstract] OR "tuberculosis"[Title/Abstract] OR "Mycobacterium tuberculosis"[Title/Abstract] OR "respiratory tract infection*"[Title/Abstract] OR "bacterial pneumonia"[Title/Abstract] OR "sepsis"[Title/Abstract] OR "respiratory virus*"[Title/Abstract] OR "viral infection"[Title/Abstract] OR "Pseudomonas aeruginosa"[Title/Abstract] OR "RSV"[Title/Abstract] OR "respiratory syncytial virus"[Title/Abstract] OR "human metapneumovirus"[Title/Abstract] OR "lower respiratory infection*"[Title/Abstract]) |
| COPD and fibrosis | #7 ("chronic obstructive pulmonary disease"[Title/Abstract] OR "COPD"[Title/Abstract] OR "pulmonary fibrosis"[Title/Abstract] OR "idiopathic pulmonary fibrosis"[Title/Abstract] OR "IPF"[Title/Abstract] OR "acute lung injury"[Title/Abstract] OR "acute respiratory distress syndrome"[Title/Abstract] OR "ARDS"[Title/Abstract] OR "asthma"[Title/Abstract] OR "emphysema"[Title/Abstract] OR "LPS"[Title/Abstract] OR "lipopolysaccharide"[Title/Abstract]) |
| Tumor | #8 ("lung neoplasm*"[Title/Abstract] OR "lung cancer"[Title/Abstract] OR "lung carcinoma"[Title/Abstract] OR "non-small cell lung cancer"[Title/Abstract] OR "NSCLC"[Title/Abstract] OR "lung adenocarcinoma"[Title/Abstract] OR "tumor microenvironment"[Title/Abstract] OR "tumor-on-chip"[Title/Abstract] OR "cancer organoid"[Title/Abstract] OR "neoplasm*"[Title/Abstract]) |
| Immunity and stromal interactions | #9 ("macrophage*"[Title/Abstract] OR "alveolar macrophage*"[Title/Abstract] OR "fibroblast*"[Title/Abstract] OR "T-lymphocyte*"[Title/Abstract] OR "T cell*"[Title/Abstract] OR "dendritic cell*"[Title/Abstract] OR "inflammation"[Title/Abstract] OR "cytokine*"[Title/Abstract] OR "cellular immunity"[Title/Abstract] OR "immune cell*"[Title/Abstract] OR "immune microenvironment"[Title/Abstract] OR "tumor-associated macrophage*"[Title/Abstract] OR "TAM"[Title/Abstract] OR "neutrophil*"[Title/Abstract]) |
| Methodology | #10 ("protocol"[Title/Abstract] OR "generation"[Title/Abstract] OR "establishment"[Title/Abstract] OR "differentiation"[Title/Abstract] OR "characterization"[Title/Abstract] OR "maturation"[Title/Abstract] OR "self-assembly"[Title/Abstract] OR "culture system*"[Title/Abstract]) |
| Combined | #11 (#1 AND #2) |
|  | #12 (#11 AND (#3 OR #4 OR #5 OR #6 OR #7 OR #8 OR #9 OR #10)) |

Note: Some foundational/cross-organoid comparative references and epidemiological background data could not be captured by the core search strategy because their subject terms do not directly overlap with "pulmonary organoids." Therefore, they were supplemented through classic literature citation tracking and independent background searches, consistent with standard review writing practices.

(1) Foundational/cross-organoid comparative references (e.g., intestinal organoids, brain organoids, gut-on-chip, etc.): identified through citation tracking of classic literature and included to describe the historical development of organoid technology in the Introduction.

(2) Epidemiological and global disease burden references (e.g., GBD 2019/2021 data, WHO reports, lung cancer/tuberculosis incidence and mortality statistics): retrieved through independent background searches (e.g., "global burden of disease" AND (lung OR tuberculosis)) and included to describe the research background and disease burden.
